# The association between dietary meal intake habits and coronary artery stenosis and cardio-metabolic risk factors

**DOI:** 10.1186/s40795-024-00895-1

**Published:** 2024-06-14

**Authors:** Marzieh Taftian, Bahareh Sasanfar, Mohammadtaghi Sarebanhassanabadi, Seyedmostafa Seyedhosseini, Sayyed Saeid Khayyatzadeh, Farzan Madadizadeh, Maryam Motallaei, Sara Beigrezaei, Faezeh Golvardi-Yazdi, Fatemeh Mirjalili, Amin Salehi-Abargouei

**Affiliations:** 1 Research Center for Food Hygiene and Safety, School of Public Health, Shahid Sadoughi University of Medical Sciences, Yazd, Iran; 2 Department of Nutrition, School of Public Health, Shahid Sadoughi University of Medical Sciences, Yazd, Iran; 3 Yazd Cardiovascular Research Center, Noncommunicable disease Research institute, Shahid Sadoughi University of Medical Sciences, Yazd, Iran; 4 Center for healthcare Data modeling, Departments of biostatistics and Epidemiology, School of public health, Shahid Sadoughi University of Medical Sciences, Yazd, Iran; 5 Department of Nursing, School of Nursing and Midwifery, Shahid Sadoughi University of Medical Sciences and Health Services, Yazd, Iran; 6 Student Research Committee, Shahid Sadoughi University of Medical Sciences, Yazd, Iran

**Keywords:** Dietary meal intake habits, Coronary artery stenosis, Anthropometric indices, Lipid profile, Fasting blood sugar, Blood pressure, Syntax score, Gensini score, Coronary artery disease

## Abstract

**Background and objective:**

We are not aware of studies examining the association between dietary meal intake habits (DMIH) and severity of coronary artery stenosis (CAS). This study was conducted to investigate the relationship between DMIH and the severity of CAS as well as cardiometabolic risk factors in adults undergoing coronary angiography.

**Methods:**

This cross-sectional study was done on 720 patients undergoing coronary angiography (aged 35–75 years) who were admitted to Afshar Hospital, a referral hospital for cardiovascular diseases in Yazd, Iran. Data on DMIH were gathered by interview. Blood samples were taken for biochemical analysis. Blood pressure, anthropometric indices, and body composition were also evaluated. The relationship between DMIH and the severity of CAS [examined by angiography based on Gensini Score (GS) and Syntax Score (SS)] and cardiometabolic risk factors were assessed using logistic regression and the analysis of covariance (ANCOVA), respectively, in crude and multivariable adjusted models.

**Results:**

After adjustment for all possible confounding variables, the study revealed that people who ate 3 meals/day had a lower risk of severe CAS compared to people who ate 2 or fewer meals (OR = 0.48, 95% CI: 0.26, 0.88, P-trend = 0.02). There was an inverse association between the number of snacks /day and the severity of CAS (OR = 0.43, 95% CI: 0.22, 0.87, P-trend = 0.02). There was also an inverse relationship between breakfast frequency/week and the severity of CAS based on both GS and SS (*P* < 0.05). Breakfast consumption, meal frequency, lunch consumption, snack frequency, and more food consumption on holidays were also associated with different cardiometabolic markers and anthropometric measures (*P* < 0.05).

**Conclusion:**

According to the results of the present study, meal frequency and breakfast consumption might be inversely associated with CAS and cardiometabolic risk factors.

## Introdution

Cardiovascular diseases (CVDs), the leading cause of global mortality, caused about 17.9 million deaths in 2019, which accounts for 32% of all global deaths [1]. The direct and indirect costs of CVD due to productivity loss in the United States are projected to rise from US$445 billion in 2010 to US$1,094 billion in 2030 [2]. The disease is also the leading cause of death for people over 35 years old in Iran [3]. The main risk factors for CVD are hypertension, high blood sugar, abdominal obesity, low HDL (High-Density-Lipoprotein) cholesterol and high serum triglycerides which are associated with unhealthy diet, physical inactivity, smoking, globalization, urbanization and population aging, poverty, stress and hereditary factors and harmful alcohol consumption [4].

Besides the dietary food and nutrient content as a key risk factor for CVDs, eating habits may also play a role. In other words, people consume a combination of foods within the framework of their eating habits [5]. Several eating habits including the number of daily meals and snacks, the consumption or non-consumption of breakfast, dinner, or lunch, and the consumption of intra-meal fluid have been studied for their effect on diseases [6–15]. Numerous research studies have indicated that dietary patterns significantly influence the risk of developing cardiovascular diseases [16–22]. For instance, it has been suggested that skipping meals, such as breakfast, can lead to cardiometabolic disorders and ultimately to CVD. Skipping lunch may also be associated with an increase in diastolic pressure [23]. It was also shown that increasing meal numbers is linked to better cardiovascular health [16, 19–26]. However, only one study examined the relationship between meal number and coronary artery stenosis, and found no association [27]. The findings of previous studies that have assessed the relationship between meal number and cardiovascular disease risk factors were also inconsistent [20, 23, 24, 28]. A 1-year prospective study of 116 US women showed that meal number was inversely associated with blood pressure [20]. On the other hand, a cross-sectional study in female hospital nurses concluded that more snacking was related to higher body fat percentage [24]. Murakami and Livingstone also indicated that meal number was positively associated with obesity and overweight [28]. However, in a cross-sectional study, meal number was inversely associated with abdominal obesity, blood pressure, and serum triglycerides [22]. On the other hand, Sanei et al. found no association between meal number and cardiovascular disease risk factors [14].

To date, very few studies have investigated dietary meal intake habits in relation to cardiovascular diseases and coronary artery disease in particular. To our knowledge, there is no data on the relationship between dietary meal intake habits and the severity of coronary artery stenosis by using an objective index. Also, the discrepancies in findings of previous studies, especially when examining the association with cardiovascular disease risk factors, motivated us to conduct a cross-sectional study to explore the relationship between dietary meal intake habits and severity of coronary artery stenosis by simultaneously measuring two objective indicators in people undergoing coronary angiography as well as cardiovascular disease risk factors.

## Methods

This cross-sectional study is reported in agreement with the recommendation of Opinion Strengthening the Reporting of Observational Studies in Epidemiology (STROBE) [29].

### Study design

This study was conducted in the context of a cross-sectional study called Iranian-CARDIO study with the aim of determining the association between lifestyle risk factors with lipid profile, fasting blood glucose, anthropometric indices, blood pressure and degree of coronary artery stenosis in subjects referred to Afshar Hospital which is a referral hospital in Yazd, Iran for coronary angiography. The detailed protocol of the Iranian-CARDIO is reported elsewhere [30]. After assessing eligibility based on inclusion and exclusion criteria using patients’ medical history, an informed consent was obtained for all participants. After that data on demographics, socioeconomic status, alcohol and tobacco use, menstrual status in women, chronic disease history, physical activity, and eating habits, were collected via interview. Blood samples were taken when the patients were in a fasted state. Anthropometric measurement was also done for all participants.

### Participants

We included adults (aged 35 to 75 years) who were referred to Afshar Hospital for coronary angiography for the first time in the Iranian-CARDIO study. Participants with the following criteria were excluded: (1) Subjects with a history of cancer, chronic heart failure, subjects with a history of MI (myocardial infarction), PCI (percutaneous coronary intervention) or CABG (coronary artery bypass graft), chronic kidney disease stage 3 and above, a specific liver disease, a specific cognitive or psychiatric disorder, immune deficiency syndrome (AIDS); (2) People with extreme obesity (BMI > 40 kg/m^2^); (3) Pregnant and lactating women; (4) Individuals with limitations in oral feeding or a special diet. In total, 735 patients met the eligibility criteria and participated in the present study between July 2020 to November 2021. Out of the 735 participants in the current study, we excluded data from 15 individuals due to a history of cancer, hepatitis, lupus, or multiple sclerosis (MS) and finally included data from 720 participants in the analyses of this study.

### Variables

#### Determining the severity of coronary artery stenosis

Angiography of the patients was performed with an angiogram device (Siemens, Axiom Artis, Germany) Subsequently, the severity of coronary artery stenosis was calculated by a cardiologist, unaware of the collected data except for age and gender, using two scoring systems: Gensini score and Syntax score. To ensure the accuracy of the results of the initial calculations of the Gensini score and the Syntax score, another cardiologist then randomly calculated the scores of several participants. The Gensini score was calculated by associating each detected coronary artery stenosis with the severity of the occlusion as follows: 1 point for stenosis ≤ 25%, 2 points for 26–50% stenosis, 4 points for 51–75% stenosis, 8 points for 76–90% stenosis, 16 points for 91–99% stenosis, and 32 points for full stenosis (100%). Thereafter, the score of each lesion was multiplied by a factor that takes into account the importance of the coronary arteries and the location of the lesion in the coronary circulation (5 for the left main coronary artery, 2.5 for the proximal left anterior descending coronary artery, 2.5 for the proximal part of the circumflex artery, 1.5 for the middle part of the left anterior descending coronary artery, 1 for the right coronary artery, the distal part of the left anterior descending coronary artery, the posterolateral artery and the single peripheral artery, and 5.0 for other sections. Finally, the Gensini score was determined from the sum of the coronary segment scores. A higher Gensini score for a person indicates greater disease severity [31–33]. Subjects were divided into two groups based on their Gensini score: those with the score of less than 20 were considered as mild to moderate coronary artery stenosis and those with the score of 20 and higher were considered as severe coronary artery stenosis [34].

The Syntax score was calculated using the second version of the internet-based Syntax calculator. The Syntax Score algorithm consists of sequential and interactive self-paced questions that focus on functional and anatomical parameters of lesions with 50% stenosis in 1.5 mm diameter arteries. The final Syntax score was determined by summing all lesion scores. Subjects with a score of less than 23 were categorized as no coronary artery stenosis or mild coronary artery stenosis and subjects with a score of at least 23 were considered as moderate to severe coronary artery stenosis [34, 35].

#### Serum lipid profile and fasting glucose assessment

Five ml of venous blood samples were collected from all participants in a fasted state and transferred equally to two separate EDTA blood tubes. One of the two blood samples was centrifuged at 2500 rpm for 3 min to separate the serum from blood cells. Commercially available kits (Biorex, Iran) were used to evaluate serum fasting blood sugar (FBS), triglycerides (TG), and total cholesterol (TC). The determination of low-density lipoprotein (LDL) and high-density lipoprotein (HDL) levels was conducted using the PARS Azmoon (Iran) and Bayerpaul (Iran) standard kits. All analyses were conducted by utilizing an auto-analyzer (Alpha Classic, AT Plus, Iran).

#### Blood pressure measurement

Blood pressure was measured before the angiography by nurses working in the cardiovascular care unit. Measurements were performed on the right arm of fully rested participants in the supine position using an NIBP module (Version 3, Sazgan Gostar, Iran) connected to a bedside monitor (Vectra, Sazgan Gostar, Iran). The systolic and diastolic blood pressures displayed on the monitor were recorded.

#### Anthropometry and Body composition measurements

To measure anthropometry, we first placed the patient’s back in a standing position and as straight as possible and attached it to the wall under a wall-mounted height meter with an accuracy of 0.1 cm. Then we measured and recorded body composition including body fat percentage, skeletal-muscle percentage, and visceral fat percentage and body weight using a bioelectrical impedance analyzer (model no. BF511, Omron, Japan) with an accuracy of 100 g, without the patient shoes, and minimal clothes. We then measured waist circumference with a non-stretchable tape measure while the patient was standing at the narrowest point in the waist, which is between the pelvic bone and the last rib above the pelvis. If the narrowest waist area was not found due to the presence of abdominal fat or excessive thinness, we measured the waist just below the last rib. The tape measure should not be too loose or tight Anthropometric measurements were performed by trained nutritionists. Body mass index was calculated by dividing weight (in kilograms) by height (in meters) squared.

#### Physical activity assessment

We assessed physical activity using the short version of the International Physical Activity Questionnaire (IPAQ), which has been validated in the Iranian population [36]. This questionnaire asks about people’s light, moderate, and vigorous physical activity in five different domains (work-related, transportation-related, housework-related, rest and leisure-related physical activity). We then converted the data for activity into metabolic equivalents of minutes per week (met min/week) [37].

#### Dietary meal intake habits

Dietary meal intake habits of the participants were examined based on previous studies using 9 multiple choice questions [6, 8, 10, 12, 14, 38] including the number of main meals (How many main meals do you eat daily? • 1 serving • 2 servings • 3 servings), the number of snacks (How many snacks do you eat daily? -Snack means eating anything outside of meals. For example, eating an apple or a piece of cake, or a glass of milk or a chocolate- • I don’t eat snacks • 1–2 snacks • 3–5 snacks • More than 5 snacks), eating on holidays (Do you eat more on holidays than normal days of the week? • never • sometimes • often • always), the regular consumption of meals (Do you eat your meals regularly? • never • sometimes • often • always), the frequency of breakfast (How many days a week do you eat breakfast? • Never or 1 day • 2–4 days • 5–6 days • Every day), breakfast consist only of liquids (Usually, how many days a week does your breakfast consist only of liquids (tea or milk)?• Never or 1 day • 2–4 days • 5–6 days • Every day), lunch (How many days a week do you usually have lunch? • Never or 1 day • 2–4 days • 5–6 days • Every day), dinner (How many days a week do you usually have dinner? • Never or 1 day • 2–4 days • 5–6 days • Every day) and evening snack (How many days a week do you usually have dinner? • Never or 1 day • 2–4 days • 5–6 days • Every day) through interviews.

#### Other variables

Gender, age and marital status were collected using a questionnaire with birth certificate information. Additionally, graduation, economic status using a socioeconomic status questionnaire, menstrual status using an associated questionnaire, smoking status and drug dependence using a history of alcohol and tobacco use questionnaire, and cardiovascular disease and type 2 diabetes using a participant medical history questionnaire were also measured questioned. Total daily energy intake was also calculated from food intake using a valid and reliable 182-item semi-quantitative multiple-choice food frequency questionnaire (FFQ).

### Statistical analysis

When describing the variables, we compared the general characteristics of the participants according to the severity of coronary artery stenosis based on the Gensini and Syntax scores, in subjects with moderate to severe coronary artery stenosis (Syntax score 23 or Gensini score 20) and subjects with mild coronary artery stenosis (Syntax score < 23 or Gensini score < 20) using independent samples t-test for quantitative variables (mean standard deviation) and chi-square test for qualitative variables (frequency and percentage). When analyzing and reviewing the reports we examined the relationship between dietary meal intake habits and the severity of coronary artery stenosis based on Gensini and Syntax scores, using multiple binary logistic regression with univariate and multivariate data after adjusting for potential confounding variables. To adjust for potential confounding variables, we used age and gender in model 1 and age, gender, economic status, physical activity, body mass index, education, menstrual status, smoking status, drug addiction, marital status, cardiovascular disease, type 2 diabetes, and energy in model 2. Then, using the analysis of covariance (ANCOVA), we assessed the relationship between each eating habit and lipid profile values, fasting blood glucose and anthropometric indices, as well as body composition (waist circumference, body fat percentage) and body mass index in two models: raw and adjusted for potential confounding variables (age, gender, economic status, physical activity, education, menstrual status, smoking status, drug addiction, marital status, cardiovascular disease, type 2 diabetes, and the energy intake). All statistical analyses were done in SPSS (Statistics Package for the Social Science) version 24 (IBM corporation, USA) and significant level was considered 0.05.

## Results

Table 1 shows the general characteristics of participants across the coronary artery stenosis. Based on the Gensini score, subjects with higher score of Gensini were older, had higher height, lower BMI, lower body fat percentage, higher skeletal muscle percentage, higher FBS, higher systolic blood pressure compared with lower score of Gensini. The frequency of male participants was more among subjects with severe coronary artery stenosis. The frequency of menstrual status, smoking status, drug addiction and diabetes type 2 were significantly different according to Gensini score status (*P* < 0.05). Based on the Syntax score, subjects with higher score of Syntax were older, had higher height, lower BMI, lower body fat percentage, higher skeletal muscle percentage compared with lower score of Syntax. The frequency of male partcipants was significantly more among subjects with moderate to severe coronary artery stenosis. The frequency of economic ststus, menstrual status, smoking status, drug addiction and diabetes type 2 were significantly different based on Syntax score status (*P* < 0.05). There were no significant differences in other variables among participants in different groups with coronary artery stenosis (*P* > 0.05).

**Table 1:** General characteristics of study participants

Variable^1^	Total Population (*n* = 720)	Coronary artery stenosis					
		Based on Gensini score		*P* value	Based on SYNTAX score		*P* value
		< 20 mild to moderate	≥ 20 sever		< 23 mild	≥ 23 moderate to severe	
**Age** ^**2**^	56.57 ± 9.78	55.03 ± 9.7	57.96 ± 9.32	**≤ 0.001**	55.88 ± 9.62	58.78 ± 9.27	**0.003**
**Weight (Kg)**	74.03 ± 12.81	73.53 ± 13.08	74.60 ± 12.45	0.299	73.88 ± 13.03	74.68 ± 11.66	0.55
**Height (Cm)**	164.06 ± 9.91	162.47 ± 10.18	165.82 ± 9.50	**≤ 0.001**	163.58 ± 10.21	166.01 ± 8.81	**0.02**
**waist circumference (Cm)**	99.96 ± 12.34	100.25 ± 13.116	99.56 ± 11.38	0.492	100.05 ± 12.42	99.40 ± 12.03	0.62
**BMI(kg / m** ^**2**^ **)**	27.53 ± 4.33	27.92 ± 4.67	27.12 ± 3.91	**0.023**	27.63 ± 4.42	27.19 ± 4.02	0.33
**Bodyfat percentage**	32.25 ± 11.04	34.79 ± 11.87	29.50 ± 9.46	**≤ 0.001**	33.15 ± 11.29	28.82 ± 9.70	**≤ 0.001**
**Skeletal muscle percentage**	30.29 ± 10.16	28.73 ± 6.18	31.82 ± 6.18	**≤ 0.001**	29.89 ± 10.84	31.35 ± 4.97	**≤ 0.001**
**Visceral fat percentage**	11.05 ± 5.19	10.77 ± 6.02	11.32 ± 4.03	0.196	10.885 ± 5.42	11.65 ± 4.09	0.16
**TC**	197.42 ± 107.27	193.74 ± 97.07	201.81 ± 115.08	0.429	199.10 ± 105.98	191.94 ± 108.65	0.58
**LDL-C**	97.77 ± 41.15	94.06 ± 38.57	101.23 ± 43.61	0.074	96.79 ± 39.73	101.305 ± 47.47	0.38
**HDL-C**	49.01 ± 13.33	50.13 ± 14.61	48.255 ± 12.11	0.150	49.23 ± 13.78	49.05 ± 11.95	0.91
**TG**	155.13 ± 85.7	152.35 ± 85.10	153.49 ± 81.61	0.886	152.88 ± 84.31	153.14 ± 79.15	0.97
**FBS**	133.24 ± 6309	126.10 ± 5906	139.45 ± 6483	**< 0.01**	130.81 ± 6212	140.01 ± 6209	0.17
**Systolic blood pressure**	128.61 ± 13.9	127.68 ± 13.66	129.59 ± 14.15	0.08	128.48 ± 14.13	129.04 ± 12.96	0.69
**Diastolic blood pressure**	85.1 ± 7174	86.94 ± 8576	84.07 ± 5729	0.628	85.65 ± 7455	85.28 ± 6985	0.96
**energy**	2652.1 ± 1565.04	2689.78 ± 1677.13	2723.52 ± 1390.99	0.780	2688.96 ± 1526.31	2804.97 ± 1635.85	0.46
**Sex** ^**3**^ **(number,%)**							
Male	61.1%	166, 47.6%	238, 76.8%	**≤ 0.001**	305,56.5%	98,83.1%	**≤ 0.001**
Female	38.3%	183, 52.4%	72, 23.2%		235,43.5%	20,16.9%	
**Economic status (%)**							
Low	30.3%	119 ,35.7%	84 ,28%	0.1	175,33.9%	28 ,24.1%	**0.04**
middle	29.9%	102 ,30.6%	99, 33%		154, 29.8%	47, 40.5%	
high	34.9%	112, 33.6%	117, 39%		187 ,36.2%	41, 35.3%	
**Physical activity (min/week)**							
Low	32.1%	118, 34.3%	102, 33.3%	0.14	187,35.2%	33,28%	0.1
Middle	32.5%	104 ,30.2%	113, 36.9%		168,31.6%	49,41.5%	
High	32.4%	122, 35.5%	91, 29.7%		177,33.3%	36,30.5%	
**degree**							
Uneducated	23.2%	89, 25.6%	62,20.1%	0.08	127,23.6%	24,20.7%	0.82
Elementary	35.6%	130, 37.5%	108,35.1%		198,36.8%	39,33.6%	
Middle school	18.3%	58, 16.7%	67,20.8%		97,18%	28,24.1%	
High school diploma	14.2	52, 15%	43,14%		79,14.7%	16,13.8%	
Associate bachelor	6.1%	16, 4.6%	24,7.8%		32,5.9%	8,6.9%	
Master	0.8%	1, 0.3%	4,1.3%		4,0.7%	1,0.9%	
Doctorate postdoctoral	0.1%	1, 0.3%	0,0%		1,0.2%	0 ,0%	
Theological	0%	0,0%	0,0%		0,0%	0,0%	
**Menstrual status**							
Yes	9.4%	54,15.4%	12,3.9%	**≤ 0.001**	166,30.7%	18,15.3%	**≤ 0.001**
No	27.2%	125,35.7%	59,19%		64,11.8%	2,1.7%	
**Smoking status**							
Non smoker	65.1%	255,72.9%	182,58.7%	**≤ 0.001**	376,69.5%	60,50.8%	**≤ 0.001**
Former smoker	2.8%	7,2%	13,4.2%		16,3%	4,3.4%	
Current smoker	32.1%	88,25.1%	115,37.1%		149,27.5%	54,45.8%	
**Drug addiction**							
non- drug addiction	77.1%	258,82.6%	229, 75.1%	**0.01**	432 ,81.1%	81,69.8%	**0.02**
former drug addiction	2.2%	10, 2.9%	6, 2%		13,2.4%	3 ,2.6%	
current drug addiction	18.1%	50 ,14.5%	70 ,23%		88,16.5%	32,27.6%	
**Marital status**							
Single	1%	2,0.6%	4,1.3%	0.62	4,0.7%	2,1.7%	0.68
married	9.1%	321,92.8%	283,91.6%		496,92.5%	107,90.7%	
Widow	6.7%	23,6.6%	22,7.1%		36,6.7%	9,7.6%	
**Cardiovascular disease**							
No	84.9%	292,84.6%	274,89.3%	0.08	461,86.2%	105,89.7%	0.3
Yes	12.5%	53,15.4%	33,10.7%		74, 13.8%	12,10.3%	
**Diabetes type 2**							
No	66.3%	251,73%	194,63.2%	**<0.01**	366,68.7%	79,66.9%	0.71
Yes	30.8%	93,27%	113,36.8%		167,31.3%	39,33.1%	

1 - T-Test was used to compare quantitative variables and Chi-square test was used for qualitative variables

2 -For quantitative variables, values are reported as mean ± standard deviation

3 -For all qualitative variables, values are reported as percentages (numbers)

Table 2 shows the relationship between dietary meal intake habits and the severity of coronary artery stenosis. The present study found that there was an inverse trend between the number of meals per day and the severity of coronary artery stenosis based on the Syntax score, and that people who ate 3 meals per day had a lower odds of severe coronary artery stenosis than people who ate two or fewer meals (OR = 0.48, 95% CI: 0.26, 0.88, P-trend = 0.02) in model 3 and after adjustment for potential confounders this association remained significant. The study also, found that there was an inverse association between the number of snacks consumed daily and the severity of coronary artery stenosis based on the Gensini score, and that people who snacked more than twice a day had less severe coronary artery stenosis based on the Gensini score than those who snacked two times or less (OR = 0.43, 95% CI: 0.22, 0.87, P-trend = 0.02). We found that there was an inverse relationship between the number of times breakfast was eaten per week and the degree of coronary artery stenosis based on the Gensini score, and that people who ate breakfast most days or every day had less severe coronary artery stenosis based on the Gensini score than those who ate breakfast four days or less (OR = 0.41, 95% CI: 0.19, 0.89, P-trend = 0.03) and the same association was observed for the Syntax score (OR = 0.34, 95% CI: 0.14, 0.79, P-trend = 0.03).

**Table 2:** Association between dietary meal intake habits and the severity of coronary artery stenosis

	Coronary artery stenosis					
Dietary habits	Based on Gensini score			Based on Syntax score		
	Crude OR (95%CI)	Model 1^1^ OR_adj_ (95%CI)	Model 2^2^ OR_adj_ (95%CI)	Crude OR (95%CI)	Model 1 OR_adj_ (95%CI)	Model 2 OR_adj_ (95%CI)
**Meal frequency** 1-How many main meals do you eat daily?						
2 meals/day or lower	1	1	1	1	1	1
3 meals/day	0.83(0.54,1.26)	**0.62 (0.39,0.99)**	0.64(0.381,1.06)	0.62(0.37,1.03)	**0.46(0.27,0.8)**	**0.48(0.26,0.88)**
*P ternd*	0.38	**0.04**	0.09	0.06	**0.01**	**0.02**
**Snack consumption** How many snacks do you eat daily? (Snack means eating anything outside of meals. For example, eating an apple or a piece of cake, or a glass of milk or a chocolate)						
Never	1	1	1	1	1	1
1–2 times/day	0.84(0.54,1.33)	0.78(0.48,1.28)	0.67(0.4,1.15)	0.87(0.49,1.54)	0.84(0.46,1.52)	0.81(0.43,1.53)
More than 2 times/day	0.61(0.34,1.08)	0.54(0.29,1)	**0.43(0.22,0.87)**	0.88(0.42,1.81)	0.81(0.38,1.75)	0.7(0.3,1.64)
*P ternd*	0.09	0.05	**0.02**	0.7	0.6	0.41
**Regular meal** Do you eat your meals regularly?						
Never	1	1	1	1	1	1
Sometimes	1.26(0.74,2.14)	1.57(0.88,2.59)	1.71(0.9,3.25)	1.52(0.75,3.07)	1.91(0.91,4.02)	1.78(0.78,4.05)
Often	0.82(0.48,1.4)	0.9(0.51,1.59)	0.97(0.51,1.83	1.28(0.62,2.64)	1.52(0.71,3.25)	1.61(0.7,3.73)
Always	1.15(0.48,1.4)	1.35(0.84,2.16)	1.34(0.79,2.3)	1.44(0.62,2.62)	1.69(0.9,3.19)	1.75(0.85,3.59)
*P ternd*	0.75	0.46	0.61	0.37	0.24	0.23
**Eat on holidays** Do you eat more on holidays than normal days of the week?						
Never	1	1	1	1	1	1
Sometimes	0.9(0.46,1.77)	0.87(0.41,1.83)	0.7(0.29,1.69)	0.81(0.33,2)	0.87(0.34,2.23)	0.73(0.23,2.28)
Often	0.66(0.29,1.49)	0.67(0.28,1.58)	0.56(0.22,1.43)	0.55(0.16,1.33)	0.58(0.17,2.05)	0.6(0.17,2.18)
Always	0.89(0.58,1.37)	0.77(0.48,1.23)	0.68(0.41,1.16)	0.73(0.4,1.33)	0.66(0.35,1.22)	0.74(0.39,1.41)
*P ternd*	0.44	0.63	0.07	0.21	0.14	0.28
**Breakfast frequency** How many days a week do you eat breakfast?						
never or 1 day	1	1	1	1	1	1
2–4 days	0.95(0.42,2.18)	0.83(0.34,2.04)	0.48(0.18,1.32)	0.71(0.27,1.85)	0.59(0.21,1.61)	0.32(0.1,1.04)
most days or everyday	0.7(0.37,1.34)	0.5(0.24,1)	**0.41(0.19,0.89)**	0.55(0.27,1.14)	**0.39(0.18,0.86)**	**0.34(0.14,0.79)**
*P ternd*	0.17	**0.02**	**0.03**	0.09	**0.01**	**0.03**
**Liquid breakfast** Usually, how many days a week does your breakfast consist only of liquids (tea or milk)?						
never or 1 day	1	1	1	1	1	1
2–4 days	0.82(0.47,1.42)	0.96(0.52,1.75)	1(0.81,1.98)	0.91(0.44,1.87)	1.04(0.49,2.21)	0.97(0.41,2.26)
most days or everyday	0.87(0.58,1.32)	0.91(0.58,1.42)	0.89(0.54,1.45)	0.82(0.47,1.42)	0.82(0.46,1.47)	0.89(0.48,1.64)
*P ternd*	0.44	0.68	0.65	0.46	0.54	0.72
**Lunch frequency** How many days a week do you usually have lunch?						
not everyday	1	1	1	1	1	1
every day	1.09(0.47,2.57)	0.98(0.4,2.39)	0.05(0.4,2.78)	1(0.33,3.03)	0.85(0.27,2.64)	0.94(0.29,3.02)
*P ternd*	0.84	0.96	0.91	0.99	0.77	0.92
**Dinner frequency** How many days a week do you usually have dinner?						
not everyday	1	1	1	1	1	1
every day	0.99(0.61,1.6)	0.67(0.39,1.14)	0.62(0.34,1.11)	1.34(0.68,2.64)	0.99(0.49,2)	0.99(0.46,2.12)
*P ternd*	0.95	0.14	0.12	0.39	0.98	0.98
**Evening snack frequency** How many days a week do you usually have dinner?						
never or 1 day	1	1	1	1	1	1
2–4 days	0.67(0.44,1.03)	**0.56(0.35,0.89)**	0.63(0.38,1.05)	0.73(0.42,1.26)	0.63(0.36,1.12)	0.58(0.31,1.08)
Most days	**0.7(0.49,0.99)**	0.7(0.48,1.03)	0.81(0.52,1.24)	0.65(0.41,1.03)	0.66(0.41,1.06)	0.61(0.36,1.04)
*P ternd*	0.04	0.07	0.31	0.06	0.08	0.06

^1^Adjusted for age and sex

^2^ Adjusted for age, gender, economic status, physical activity, body mass index, education, menstrual status, smoking status, drug addiction, marital status, cardiovascular diseases, type 2 diabetes and energy intake

Table 3 shows the relationship between dietary meal intake habits and lipid profile and fasting blood glucose. This study found that people who ate breakfast two to four days a week had a lower blood low density lipoproteins (LDL) levels (*P* = 0.03) than people who ate it most days or every day, and people who ate breakfast two to four days a week had lower blood LDL levels (*P* = 0.02) than people who ate it most days or every day. We observed that people who ate breakfast two to four days a week had significantly lower blood HDL levels than people who never ate it or only ate it for one day (*P* < 0.05).

**Table 3:** Relationship between dietary meal intake habits and lipid profile and fasting blood glucose

Dietary habits	Total cholesterol		Triglyceride		Low density lipoprotein cholesterol		High density lipoprotein cholesterol		FBS	
	Crude Mean ± SE	Adjusted^1^ Mean ± SE	Crude Mean ± SE	Adjusted^1^ Mean ± SE	Crude Mean ± SE	Adjusted^1^ Mean ± SE	Crude Mean ± SE	Adjusted^1^ Mean ± SE	Crude Mean ± SE	Adjusted^1^ Mean ± SE
Meal frequency 1-How many main meals do you eat daily?										
2 meals/day or lower	202.97 ± 13.36	207.12 ± 14.09	158.46 ± 10.53	154.21 ± 10.3	98.93 ± 5.15	97.9 ± 5.43	48.64 ± 0.7	48.31 ± 1.64	130.8 ± 6.64	136.16 ± 6.51
3 meals/day	196.87 ± 5.61	197.55 ± 5.92	15.61 ± 4.42	154.65 ± 4.33	97.79 ± 2.17	97.47 ± 2.29	48.94 ± 0.7	48.34 ± 0.69	134.24 ± 2.82	131.35 ± 2.78
P value	0.67	0.54	0.8	0.97	0.84	0.94	0.86	0.98	0.63	0.5
**Snack consumption** How many snacks do you eat daily? (Snack means eating anything outside of meals. For example, eating an apple or a piece of cake, or a glass of milk or a chocolate)										
Never	192.46 ± 12.87	197.32 ± 13.62	159.22 ± 10.31	162.41 ± 9.93	96 ± 4.88	94.13 ± 5.16	48.31 ± 1.6	47.47 ± 1.59	127.07 ± 0.02	127.78 ± 6.85
1–2 times/day	200.73 ± 6.27	200.59 ± 6.63	160.22 ± 5.01	156.52 ± 4.82	100.34 ± 2.4	99.41 ± 2.54	49.51 ± 0.78	48.74 ± 0.78	134.97 ± 3.1	132.34 ± 3.08
More than 2 times/day	191.79 ± 12.69	196.06 ± 14.29	140.46 ± 10.46	138.72 ± 10.52	88.01 ± 5.06	91.54 ± 5.59	46.9 ± 1.62	47.53 ± 1.67	133.52 ± 6.43	134.6 ± 6.44
P value	0.74	0.94	0.23	0.23	0.8	0.34	0.32	0.67	0.59	0.76
**Regular meal** Do you eat your meals regularly?										
Never	206.7 ± 13.07	217.13 ± 14.21	144.57 ± 10.38	146.81 ± 10.25	95.81 ± 4.98	96.38 ± 5.39	47.28 ± 1.56	47.75 ± 1.58	131.66 ± 6.34	133.37 ± 6.37
Sometimes	207.72 ± 12.21	199.5 ± 12.91	160.94 ± 9.71	153.37 ± 9.39	96.55 ± 4.65	94.35 ± 4.94	50.37 ± 1.48	47.4 ± 1.46	134.95 ± 6.13	135.67 ± 6.04
Often	196.83 ± 11.99	201.19 ± 12.57	144.33 ± 9.59	143.93 ± 9.15	102.33 ± 4.68	103.49 ± 4.87	48.36 ± 1.47	48.12 ± 1.43	127.32 ± 6.07	131.17 ± 5.93
Always	190.26 ± 7.53	191.14 ± 7.94	163.35 ± 6.03	160.73 ± 5.8	97.19 ± 2.91	96.41 ± 3.06	48.8 ± 0.92	48.61 ± 0.9	136.48 ± 3.82	131.54 ± 3.78
P value	0.55	0.46	0.22	0.39	0.75	0.56	0.54	0.9	0.57	0.94
**Eat on holidays** Do you eat more on holidays than normal days of the week? • never • sometimes • often • always										
Never	197.46 ± 6.10	202.84 ± 6.45	157.58 ± 4.87	156.2 ± 4.69	98.48 ± 2.33	98.31 ± 2.47	48.89 ± 0.75	47.87 ± 0.74	134.45 ± 3.05	134.17 ± 3.01
Sometimes	199.28 ± 21.82	164.18 ± 23.51	145.4 ± 17.47	124.62 ± 17.1	92.64 ± 8.23	84.77 ± 8.89	47.86 ± 2.66	46.64 ± 2.69	155.85 ± 10.51	147.06 ± 10.96
Often	192.19 ± 25.72	184.07 ± 26.66	170.19 ± 20.58	168.29 ± 19.39	88.02 ± 9.98	85.77 ± 10.49	46.59 ± 3.23	48.74 ± 3.17	124.96 ± 12.79	128.32 ± 12.13
Always	199.23 ± 12.69	197.43 ± 13.46	152.81 ± 10.22	152.11 ± 9.88	98.23 ± 4.96	99.84 ± 5.27	49.46 ± 1.58	50.36 ± 1.58	125.58 ± 6.43	120.39 ± 6.41
P value	0.99	0.42	0.79	0.29	0.7	0.32	0.85	0.49	0.09	0.12
**Breakfast frequency** How many days a week do you eat breakfast?										
never or 1 day	211.58 ± 21.23	216.71 ± 21.73	148.58 ± 17.05	150.2 ± 15.9	91 ± 8.38	92.97 ± 8.56	53.2 ± 2.61	52.5 ± 2.51	131.99 ± 10.38	144.87 ± 10.13
2–4 days	164.87 ± 17.11	162.99 ± 19.2	132.43 ± 13.58	126.56 ± 13.84	83.89 ± 6.49	79.63 ± 7.26	44.6 ± 2.084	43.47 ± 2.18	121.56 ± 8.79	122.74 ± 8.99
most days or everyday	200.2 ± 5.61	201.12 ± 5.89	160.1 ± 4.51	157.75 ± 4.32	99.68 ± 2.17	99.69 ± 2.27	49.13 ± 0.7	48.56 ± 0.69	135.24 ± 2.82	132.23 ± 2.78
P value	0.12	0.12	0.14	0.1	0.07	**0.03**	**0.03**	**0.02**	0.33	0.26
**Liquid breakfast** Usually, how many days a week does your breakfast consist only of liquids (tea or milk)?										
never or 1 day	202.73 ± 6.18	201.89 ± 6.51	155.66 ± 4.95	153.77 ± 4.76	97.85 ± 2.39	96.64 ± 2.51	49.46 ± 0.77	48.93 ± 0.75	134.14 ± 3.07	131.03 ± 3.02
2–4 days	177.77 ± 16.99	181.31 ± 18.47	171.94 ± 13.64	154.55 ± 13.51	96.06 ± 6.46	93.89 ± 6.99	46.45 ± 2.09	45.19 ± 2.12	127.84 ± 8.48	128.95 ± 8.61
most days or everyday	190.66 ± 11.8	198.09 ± 12.37	153.64 ± 9.47	157.25 ± 9.05	98.43 ± 4.59	101.7 ± 4.81	48.36 ± 1.49	48.09 ± 1.46	134.48 ± 6.11	136.96 ± 5.93
P value	0.3	0.57	0.5	0.94	0.95	0.57	0.37	0.25	0.77	0.63
**Lunch frequency** How many days a week do you usually have lunch?										
not everyday	221.42 ± 31.31	219.8 ± 31.04	154.92 ± 25.16	159.41 ± 22.73	115 ± 11.87	112.05 ± 11.76	47.41 ± 3.87	46.58 ± 3.59	134.55 ± 14.32	136.57 ± 13.28
every day	196.87 ± 5.25	198.17 ± 5.5	156.81 ± 4.21	154.34 ± 4.03	97.43 ± 2.03	97.05 ± 2.13	48.99 ± 0.65	48.44 ± 0.64	133.78 ± 2.64	132.06 ± 2.6
P value	0.44	0.49	0.94	0.83	0.14	0.21	0.69	0.61	0.96	0.74
**Dinner frequency** How many days a week do you usually have dinner?										
not everyday	207.45 ± 15.51	205.38 ± 16.11	156.82 ± 12.44	151.13 ± 11.76	102.96 ± 6	102.08 ± 6.26	45.82 ± 1.93	45.72 ± 1.88	127.85 ± 7.71	133.34 ± 7.43
every day	196.69 ± 5.5	198.41 ± 5.8	156.5 ± 4.4	154.67 ± 4.24	97.31 ± 2.12	96.94 ± 2.24	49.36 ± 0.68	48.77 ± 0.67	134.51 ± 2.77	132.01 ± 2.74
P value	0.51	0.69	0.98	0.78	0.37	0.44	0.8	0.13	0.42	0.87
**Evening snak** How many days a week do you usually have dinner?										
never or 1 day	214.22 ± 7.89	212.93 ± 8.4	160.43 ± 6.35	156.82 ± 6.15	97.91 ± 3.06	96.04 ± 3.25	49.98 ± 0.98	48.51 ± 0.98	132.16 ± 4.04	128.08 ± 4.05
2–4 days	188.09 ± 7.89	187.72 ± 11.86	143.81 ± 9.18	139.72 ± 8.68	98 ± 4.38	98.36 ± 4.54	47.62 ± 1.42	47.84 ± 1.39	137.73 ± 5.71	138.7 ± 5.59
Most days	184.23 ± 8.51	189.58 ± 9.05	159.95 ± 95 ± 6.86	160.04 ± 6.62	97.75 ± 3.36	98.69 ± 3.57	48.48 ± 1.08	48.58 ± 1.08	133.48 ± 4.28	132.92 ± 4.21
P value	0.02	0.1	0.28	0.16	1	0.84	0.34	0.9	0.72	0.3

^1^ Adjusted for age, gender, economic status, physical activity, body mass index, education, menstrual status, smoking status, drug addiction, marital status, cardiovascular diseases, type 2 diabetes and energy intake

Table 4 shows the relationship between dietary meal intake habits and body composition markers. The results indicated that people who ate two or fewer meals per day had a larger waist circumference than those who ate three or more meals (*P* = 0.04). For people who ate lunch every day of the week versus those who ate nothing, weight (*P* = 0.03), body mass index (*P* = 0.02), and waist circumference (*P* = 0.02) were significantly smaller. Also, those who ate evening snack 2 to 4 days a week had significantly smaller waist circumferences than those who ate most days (*P* = 0.04) or those who never ate evening snack (*P* = 0.01). In addition, these individuals weighed significantly less than those who ate evening snack most days (*P* = 0.04). People who always consumed more food on holidays compared to normal days compared to people who never had this behavior (*P* = 0.03) had a significantly higher body mass index, and their body fat percentage was also significantly higher (*P* = 0.04).

**Table 4:** Association between dietary meal intake habits and body composition markers

Dietary habits	Body weigh		BMI		Waist circumference		Body fat percent	
	Crude	Adjusted^1^	Crude	Adjusted	Crude	Adjusted	Crude	Adjusted
Meal frequency 1-How many main meals do you eat daily?								
2 meals/day or lower	73.88 ± 1.28	75.05 ± 1.2	27.9 ± 0.44	28.06 ± 0.45	101.41 ± 1.26	102.56 ± 1.35	33.93 ± 1.13	33.41 ± 0.81
3 meals/day	73.97 ± 0.56	73.74 ± 0.56	27.43 ± 0.19	27.37 ± 0.19	99.59 ± 0.54	99.48 ± 0.57	31.95 ± 0.48	31.96 ± 0.34
*P value*	0.95	0.35	0.32	0.16	0.18	**0.04**	0.11	0.1
**Snack consumption** How many snacks do you eat daily? (Snack means eating anything outside of meals. For example, eating an apple or a piece of cake, or a glass of milk or a chocolate)								
Never	71.93 ± 1.34	72.97 ± 1.34	27.17 ± 0.46	27.19 ± 0.46	99.52 ± 1.3	99.43 ± 1.38	31.97 ± 1.19	31.47 ± 0.84
1–2 times/day	74.26 ± 0.61	74.19 ± 0.62	27.59 ± 0.21	27.57 ± 0.21	100.14 ± 0.6	100.26 ± 0.64	32.17 ± 0.53	32.21 ± 0.38
More than 2 times/day	74.51 ± 1.27	73.88 ± 1.27	27.4 ± 0.43	27.4 ± 0.44	99.13 ± 1.24	99.31 ± 1.32	32.77 ± 1.11	32.7 ± 0.78
*P value*	0.26	0.71	0.68	0.74	0.73	0.73	0.86	0.56
**Regular meal** Do you eat your meals regularly?								
Never	73.61 ± 1.24	73.44 ± 1.27	27.26 ± 0.42	27.38 ± 0.44	99.45 ± 1.22	99.62 ± 1.33	31.26 ± 1.08	32.09 ± 0.78
Sometimes	74.42 ± 1.22	74.24 ± 1.22	27.79 ± 0.41	27.82 ± 0.42	98.73 ± 1.19	98.9 ± 1.27	32.62 ± 1.06	32.6 ± 0.75
Often	73.46 ± 1.22	73.1 ± 1.22	27.33 ± 0.41	27.3 ± 0.42	99.82 ± 1.19	99.97 ± 1.26	32.1 ± 1.08	32.17 ± 0.77
Always	74.03 ± 0.75	74.26 ± 0.75	27.55 ± 0.25	27.47 ± 0.26	100.57 ± 0.72	100.58 ± 0.77	32.61 ± 0.65	32.18 ± 0.46
*P value*	0.94	0.83	0.79	0.83	0.58	0.71	0.73	0.96
***Eat on holidays*** Do you eat more on holidays than normal days of the week? • never • sometimes • often • always								
Never	72.98 ± 0.59	73.28 ± 0.6	27.33 ± 0.2	27.22 ± 0.21	99.56 ± 0.58	99.58 ± 0.62	32.22 ± 0.52	31.66 ± 0.37
Sometimes	74.79 ± 2.45	74.72 ± 2.24	28.06 ± 0.72	28.37 ± 0.77	101.04 ± 2.07	101.8 ± 2.31	33.11 ± 1.8	34.31 ± 1.36
Often	73.34 ± 2.45	71.18 ± 2.42	26.04 ± 0.84	26.19 ± 0.83	95.74 ± 2.39	96.17 ± 2.5	29.13 ± 2.17	30.55 ± 1.51
Always	78.09 ± 1.27	77 ± 1.27	28.38 ± 0.43	28.59 ± 0.44	101.6 ± 1.23	101.71 ± 1.3	32.47 ± 1.11	33.99 ± 0.78
*P value*	**0.00**	0.4	**0.04**	**0.01**	0.14	0.16	0.51	**0.01**
**Breakfast frequency** How many days a week do you eat breakfast?								
never or 1 day	71.4 ± 2	72.21 ± 2.05	27.37 ± 0.68	27.6 ± 0.71	98.41 ± 1.94	99.76 ± 2.11	33.4 ± 1.73	32.95 ± 1.25
12 − 4 days	74.95 ± 1.74	75.3 ± 1.79	27.19 ± 0.89	27.57 ± 0.62	101.18 ± 1.72	101.74 ± 1.87	31.6 ± 1.54	32.5 ± 1.11
most days or everyday	74.08 ± 0.55	73.97 ± 0.56	27.56 ± 0.19	27.48 ± 0.19	99.9 ± 0.54	99.85 ± 0.57	32.23 ± 0.49	32.11 ± 0.34
*P value*	0.37	0.52	0.82	0.98	0.56	0.63	0.73	0.78
**Liquid breakfast** Usually, how many days a week does your breakfast consist only of liquids (tea or milk)? •								
never or 1 day	74.5 ± 0.6	74.35 ± 0.6	27.65 ± 0.2	27.64 ± 0.21	100.54 ± 0.58	100.56 ± 0.62	32.31 ± 0.52	32.35 ± 0.36
2–4 days	73.43 ± 1.7	73.37 ± 1.75	26.92 ± 0.58	26.83 ± 0.61	99.06 ± 1.69	99.47 ± 1.83	31.84 ± 1.46	31.63 ± 1.04
most days or everyday	72.27 ± 1.21	72.83 ± 1.19	27.16 ± 0.41	27.12 ± 0.41	97.72 ± 1.17	97.95 ± 1.22	32.35 ± 1.07	32.03 ± 0.72
*P value*	0.24	0.49	0.33	0.29	0.09	0.16	0.95	0.77
**Lunch frequency** How many days a week do you usually have lunch?								
not everyday	79.75 ± 2.72	79.67 ± 2.63	29.43 ± 0.92	29.62 ± 0.9	105.59 ± 2.64	105.99 ± 2.69	31.54 ± 2.36	31.95 ± 1.61
every day	73.79 ± 0.52	73.67 ± 0.52	27.44 ± 0.18	27.4 ± 0.18	99.7 ± 0.51	99.76 ± 0.54	32.67 ± 0.45	32.19 ± 0.32
*P value*	**0.03**	**0.03**	**0.03**	**0.02**	**0.03**	**0.02**	0.76	0.88
**Dinner frequency** How many days a week do you usually have dinner?								
not everyday	74.66 ± 1.49	75.33 ± 1.49	27.88 ± 0.5	27.62 ± 0.51	99.52 ± 1.45	99.87 ± 1.55	34.31 ± 1.31	32.26 ± 0.93
every day	73.95 ± 0.54	73.85 ± 0.54	27.47 ± 0.18	27.48 ± 0.19	99.96 ± 0.53	100.02 ± 0.57	32.01 ± 0.47	32.21 ± 0.33
*P value*	0.66	0.35	0.45	0.8	0.78	0.93	0.1	0.96
**Evening snack** How many days a week do you usually have evening snack ?								
never or 1 day	73.04 ± 0.79	73.46 ± 0.81	27.4 ± 0.27	27.42 ± 0.28	100.35 ± 0.77	100.42 ± 0.83	32.28 ± 0.69	32.01 ± 0.5
2–4 days	73.05 ± 1.12	72.06 ± 1.11	26.91 ± 0.38	26.94 ± 0.39	97.26 ± 1.09	96.97 ± 1.14	30.68 ± 0.98	31.77 ± 0.7
Most days	75.45 ± 0.83	75.55 ± 0.84	27.91 ± 0.28	27.84 ± 0.29	100.86 ± 0.81	101.18 ± 0.88	33.07 ± 0.72	32.55 ± 0.52
*P value*	0.07	**0.04**	0.1	0.18	0.02	**0.01**	0.15	0.62

^1^Adjusted for age, gender, economic status, physical activity, education, menstrual status, smoking status, drug addiction, marital status, cardiovascular diseases, type 2 diabetes and energy intake

Table 5 shows the relationship between dietary meal intake habits and blood pressure. No significant association was observed between dietary meal intake habits and systolic and diastolic blood pressure (*P* > 0.05).

**Table 5:** The relationship between dietary meal intake habits and blood pressure

Dietary habits	Systolic blood pressure		Diastolic blood pressure	
	Crude(Mean ± SE)	Adjusted^1^ (Mean ± SE)	Crude(Mean ± SE)	Adjusted (Mean ± SE)
Meal frequency How many main meals do you eat daily?				
2 meals/day or lower	126.09 ± 1.37	126.62 ± 1.48	84.47 ± 7.29	86.31 ± 7.57
3 meals/day	128.98 ± 0.59	129.24 ± 0.64	85.68 ± 3.15	85 ± 3.28
*P value*	**0.05**	0.11	0.88	0.87
**Snack consumption** How many snacks do you eat daily? (Snack means eating anything outside of meals For example, eating an apple or a piece of cake, or a glass of milk or a chocolate)				
Never	127.7 ± 1.45	128.3 ± 1.55	79.01 ± 7.67	79.94 ± 7.89
1–2 times/day	128.43 ± 0.56	128.77 ± 0.71	84.44 ± 3.46	85.25 ± 3.64
More than 2 times/day	129.47 ± 1.35	129.25 ± 1.47	95.4 ± 7.117	89.65 ± 7.51
*P value*	0.66	0.91	0.26	0.68
**Regular meal** Do you eat your meals regularly?				
Never	127.92 ± 1.33	128.23 ± 1.45	86.22 ± 7.07	86.29 ± 7.4
Sometimes	127.81 ± 1.32	127.82 ± 1.41	87.83 ± 7.11	80.56 ± 7.21
Often	127.67 ± 1.31	128.25 ± 1.4	84.47 ± 7.01	84.77 ± 7.14
Always	129.4 ± 0.79	129.72 ± 0.87	84.93 ± 4.23	86.83 ± 4.43
*P value*	0.54	0.6	0.98	0.9
***Eat on holidays*** Do you eat more on holidays than normal days of the week? *• never • sometimes • often • always*				
Never	128.36 ± 0.64	128.94 ± 0.69	81.09 ± 3.37	82.19 ± 3.54
Sometimes	133.03 ± 2.75	132.2 ± 2.58	125.59 ± 11.92	105.47 ± 13.16
Often	123.76 ± 2.75	125.13 ± 2.86	77.6 ± 14.5	76.09 ± 14.59
Always	129.19 ± 1.35	128.65 ± 1.48	93.4 ± 7.14	95.28 ± 7.54
*P value*	0.65	0.33	**0.002**	0.16
**Breakfast frequency** How many days a week do you eat breakfast?				
never or 1 day	124.83 ± 2.15	125.92 ± 2.31	93.73 ± 11.4	98.26 ± 11.78
12 − 4 days	130.09 ± 1.87	129.94 ± 2.08	80.78 ± 9.94	79.38 ± 10.62
most days or everyday	128.68 ± 0.59	128.97 ± 0.64	85.36 ± 3.15	84.81 ± 3.28
*P value*	0.16	0.38	0.69	0.46
**Liquid breakfast** Usually, how many days a week does your breakfast consist only of liquids (tea or milk)?				
never or 1 day	128.69 ± 0.64	128.75 ± 0.7	86.17 ± 3.42	85.56 ± 3.56
2–4 days	128.52 ± 1.84	129.9 ± 2.03	81.68 ± 9.78	80.19 ± 10.39
most days or everyday	128.23 ± 1.23	128.98 ± 1.36	84.9 ± 6.83	86.43 ± 6.96
*P value*	0.95	0.87	0.91	0.87
**Lunch frequency** How many days a week do you usually have lunch?				
not everyday	128.39 ± 2.87	128.75 ± 2.99	79.13 ± 15.22	75.65 ± 15.25
every day	128.57 ± 0.56	128.87 ± 0.6	85.75 ± 2.95	85.66 ± 3.07
*P value*	0.95	0.97	0.67	0.52
**Dinner frequency** How many days a week do you usually have dinner?				
not everyday	126.4 ± 1.59	127.92 ± 1.71	76.8 ± 8.43	77.63 ± 8.7
every day	128.84 ± 0.58	128.98 ± 0.63	86.67 ± 3.09	86.33 ± 3.22
*P value*	0.15	0.57	0.27	0.35
**Evening snack** How many days a week do you usually have evening snack?				
never or 1 day	128.69 ± 0.85	128.85 ± 0.94	86.95 ± 4.553	88.06 ± 4.78
2–4 days	126.69 ± 1.2	126.81 ± 1.29	82.26 ± 6.34	83.69 ± 6.61
Most days	129.35 ± 0.9	129.93 ± 0.97	85.79 ± 4.75	83.07 ± 4.97
*P value*	0.2	0.16	0.83	0.74

^1^Adjusted for age, gender, economic status, physical activity, body mass index, education, menstrual status, smoking status, drug addiction, marital status, cardiovascular diseases, type 2 diabetes and energy intake

## Discussion

This study found that an increase in the number of meals eaten daily might be associated with a decrease in the severity of coronary artery stenosis based on the Syntax score. Furthermore, an increase in the number of snacks eaten daily was associated with a decrease in the severity of coronary artery stenosis based on the Gensini score. It was also observed that people who ate breakfast most days or every day had a significantly lower chance of suffering from severe artery stenosis.

To date, we are not aware of any study assessing the correlation between dietary meal intake habits and the severity of coronary artery stenosis using an objective index. Nevertheless, various studies have demonstrated a positive impact of increased meal frequency on cardiovascular health when investigating the link between meal frequency and cardiovascular or cardiometabolic diseases [16, 17, 19–23, 25, 26, 39]. Furthermore, only one population-based prospective longitudinal cohort study of 13,328 adults in Canada examined the relationship between eating habits and the mortality and hospitalization rates due to coronary artery stenosis, and in the end, no significant relationship could be found [27].

In this study, people who ate breakfast two to four days per week had a significantly lower blood HDL levels than those who ate less, these people had also a lower blood LDL levels than people who ate most days or every day.

Similar to our results a prospective case-controlled study conducted in western India with 1607 participants revealed that individuals who skip breakfast face higher risk of developing coronary artery disease and high blood pressure [40]. Skipping breakfast negatively impacts the circadian clock and is associated with an increased postprandial glycemic response [41]. Various hypotheses have been proposed about how skipping breakfast can lead to cardiometabolic disorders, which can ultimately lead to cardiovascular disease [1]. In summary, regular breakfast eaters who skip breakfast compensate by eating more later in the day. In addition, eating breakfast beforehand affects metabolic and endocrine responses to the foods eaten during the day [42]. It is also shown that eating a regular breakfast increases satiety, thus reducing overeating later in the day and preventing weight gain. Skipping breakfast enhances the risk of obesity, metabolic syndrome, high blood pressure, high cholesterol, type 2 diabetes (T2DM), coronary artery disease (CAD), and cardiovascular and all-cause mortality. Furthermore, those who skip breakfast tend to consume refined and sweet products, possibly more at night, increasing the risk of cardiovascular disease and type 2 diabetes. Skipping breakfast increases oxidative stress and inflammation, which can damage adipocytes, pancreatic beta cells, endothelial and smooth muscle cells, and neurons, leading to dysfunction of these cells and associated diseases [43].

We found that people who ate two or fewer meals a day had a larger waist circumference than those who ate three or more meals. Weight, body mass index and waist circumference were significantly lower in people who ate lunch every day of the week compared to those who did not eat lunch every day. Also, those who had evening meals for 2–4 days had significantly smaller waist circumferences than those who had most days or never had, and these individuals also weighed significantly less than those who had evening meals for most days. Also, people who always consumed more food on holidays compared to normal days compared to people who never had this behavior had a significantly higher body mass index, and their body fat percentage was also significantly higher. In line with our results, it is shown that skipping lunch may be associated with an increase in diastolic pressure [23]. In one study, late dinner eating was shown to be associated with an increased incidence of cardiovascular disease [17]. However, in general, skipping dinner was associated with increases in triglyceride levels and in the total cholesterol to low-density lipoprotein ratio, and has potential implications for cardiovascular disease risk [23]. In a cross-sectional study conducted on 73 nurses, it was found that neither total energy intake nor macronutrient consumption, but rather dietary behavior (i.e., more frequent snacking and greater variety in daily energy intake) were significantly associated with indicators of cardiometabolic health, including BMI, body fat percentage, and waist circumference [24]. A study conducted in this regard showed that the number of meals was positively related to obesity and overweight [28]. A study examining the association between the number and regularity of meals, and the frequency of dinner and lunch per week in obesity, similar to our study, failed to find a significant association in many cases, and only found that more irregular meal consumption increased the likelihood of general obesity and abdominal obesity [14]. Another cross-sectional study investigating the association between Eating Frequency EF, Meal Frequency (MF), and Snacking Frequency (SF) and Overweight/Obese and Central Obesity showed that higher EF, MF, and SF increased the odds of Overweight/Obese and Central Obesity among adults in the USA [28].

### Strengths of the study

According to our knowledge, this study is the first study with a relatively large sample size which investigated the association between dietary meal intake habits and the severity of coronary artery stenosis by measuring two objective factors. Furthermore, two reliable scoring systems have been utilized to evaluate the severity of coronary artery disease. We tried to include new cases to better examine the causal associations. A wide range of possible confounders was examined in this study, including age, gender, economic status, physical activity level, body mass index, education and menstrual status, smoking status, drug addiction, marital status, cardiovascular disease, type 2 diabetes and energy intake were adjusted.

### Limitations of the study

The cross-sectional design of this study precludes demonstrating a causal relationship between dietary meal intake habits and the severity of coronary artery stenosis as well as lipid indices, blood glucose and body composition. Therefore, further prospective studies in this area are needed. It was not possible to assess other confounding factors that might affect our results. It should also be noted that this study is not free from memory and recall errors. We were unable to assess the validity and reliability of our questionnaire. Therefore, designing a valid questionnaire to assess dietary meal intake habits is recommended.

## Conclusion

In conclusion the current study provided evidence regarding the association between dietary meal intake habits and the severity of coronary artery stenosis and cardiometabolic health. In particular, meal frequency and breakfast consumption were inversely associated with stenosis and cardiometabolic risk factors. Prospective cohort studies should be conducted to confirm these findings.

## Data Availability

The complete data set derived from this investigation is readily accessible to the corresponding author The data shall be made accessible to all researchers who are interested in the subject matter upon request from the principal investigator (ASA).
